# Overview of Aerosolized Florida Red Tide Toxins: Exposures and Effects

**DOI:** 10.1289/ehp.7501

**Published:** 2005-02-10

**Authors:** Lora E. Fleming, Lorraine C. Backer, Daniel G. Baden

**Affiliations:** ^1^National Institute of Environmental Health Sciences Marine and Freshwater Biomedical Sciences Center and National Science Foundation–National Institute of Environmental Health Sciences Oceans and Human Health Center, University of Miami Rosenstiel School of Marine and Atmospheric Sciences, Miami, Florida, USA;; ^2^National Center for Environmental Health, Centers for Disease Control and Prevention, Atlanta, Georgia, USA;; ^3^Center for Marine Science, University of North Carolina at Wilmington, Wilmington, North Carolina, USA

**Keywords:** brevetoxins, harmful algal blooms (HABs), *Karenia brevis*, red tides, sensitive populations

## Abstract

Florida red tide is caused by *Karenia brevis*, a dinoflagellate that periodically blooms, releasing its potent neurotoxin, brevetoxin, into the surrounding waters and air along the coast of the Gulf of Mexico. Exposure to Florida red tide toxins has been associated with adverse human health effects and massive fish and marine mammal deaths. The articles in this mini-monograph describe the ongoing interdisciplinary and interagency research program that characterizes the exposures and health effects of aerosolized Florida red tide toxins (brevetoxins). The interdisciplinary research program uses animal models and laboratory studies to develop hypotheses and apply these findings to *in situ* human exposures. Our ultimate goal is to develop appropriate prevention measures and medical interventions to mitigate or prevent adverse health effects from exposure to complex mixtures of aerosolized red tide toxins.

Algae and similar organisms are ubiquitous in aqueous environments. Some of these algae will periodically experience exuberant growth, or “blooms.” When algal blooms adversely affect people or the environment, they are called harmful algal blooms (HABs). Researchers who study the interactions of oceans and human health have identified HABs as one of the key threats to human health (Knap et al. 2002; National Research Council 1999; Sandifer et al. 2004; Tyson et al. 2004; Van Dolah 2000).

*Karenia brevis* (formerly known as *Gymnodinium breve* and *Ptychodiscus brevis*) and related species are dinoflagellates that can form HABs known as “Florida red tides.” Arrival of these HABs, often more green, brown, or dark-colored than red, is an annual event in the Gulf of Mexico, and these blooms have been observed as far north as North Carolina. *K. brevis* blooms are associated with the production of a group of powerful natural neurotoxins known as brevetoxins. As a group, the brevetoxins are lipid-soluble, trans-syn chains of multiring polyethers with molecular weights around 900 Da. Brevetoxins are depolarizing substances that open voltage-gated sodium ion channels in cell membranes, leading to uncontrolled sodium influx into the cell (Baden 1983; Purkerson et al. 1999). At least 10 different brevetoxins have been isolated in seawater blooms and *K. brevis* cultures. In addition, many analogs and derivatives produced by the organism itself, as well as metabolites produced by shellfish and other organisms in the marine food web, have been identified (Baden and Trainer 1993; Baden et al. 1995; Mattei et al. 1999; Mazumder et al. 1997; Morohashi et al. 1999; Pierce and Kirkpatrick 2001).

Although a considerable body of research exists on the organism and its toxins, relatively little exploration of exposure to and adverse health effects from *K. brevis* and the brevetoxins has been done in humans and other animals (Kirkpatrick et al. 2004; Music et al. 1973; Steidinger 1983; Steidinger and Baden 1984; Steidinger and Ingle 1972; Woodcock 1948). Blooms of brevetoxin-producing *K. brevis* have been associated with massive fish kills as well as the deaths of marine mammals (including endangered Florida manatees and dolphins) and marine birds. People who eat shellfish contaminated with brevetoxins can experience neurotoxic shellfish poisoning (NSP), a disease with acute gastrointestinal and neurologic symptoms that apparently resolve within a few days of onset (Kirkpatrick et al. 2004; Music et al. 1973; Woodcock 1948).

In addition to the cases of NSP caused by ingesting brevetoxin-contaminated shellfish, environmental exposures can cause adverse human health effects through other routes of exposure (Kirkpatrick et al. 2004; Music et al. 1973; Steidinger 1983; Steidinger and Baden 1984; Steidinger and Ingle 1972; Woodcock 1948). The *K. brevis* organism is quite fragile and is readily broken up in the surf, releasing brevetoxins into marine waters. At the air–sea interface, brevetoxin/sea water aerosols form and, upon exposure to ultraviolet light, may be modified into different mixtures of brevetoxins and/or other biologically active compounds (Pierce et al. in press). These toxin-containing aerosols affect people living, working, and visiting the affected coastal areas. In fact, people living and working in or near coastal areas have complained for years about upper and lower respiratory irritation associated with onshore winds and aerosol exposures during active Florida red tides (Baden et al. 1995; Music et al. 1973; Woodcock 1948). Woodcock (1948) was the first to document the importance of the Florida red tide toxins as an aerosol exposure. More recently, the interdisciplinary research group has verified the occurrence of self-reported upper and lower respiratory symptoms associated with inhaling brevetoxin aerosols during red tide events (Backer et al. 2003).

## Aerosolized Florida Red Tide Research Potential

Compared with many other HAB organisms, their toxins, and their possible associated human and animal diseases, aerosolized Florida red tide toxins offer many opportunities to conduct interdisciplinary, synergistic research. Current research efforts are building on more than 30 years of research characterizing the *K. brevis* organism and brevetoxins (e.g., Baden and Trainer 1993; Baden et al. 1994, 1995; Kirkpatrick et al. 2004; Music et al. 1973; Steidinger 1983; Steidinger and Baden 1984; Steidinger and Ingle 1972; Woodcock 1948). Previous research established the structure, function, and behavior of the dinoflagellate, as well as the chemical structure of the brevetoxins and a synthetic antagonist (Purkerson et al. 1999; Purkerson-Parker et al. 2000). When the neurologic activities of brevetoxins were identified, researchers began to understand the neurophysiology and the mechanisms of actions of the sodium channels in nerve cells (Purkerson et al. 1999; Trainer et al. 1991). Advances in molecular biochemistry, in turn, pushed the limits of the analytic measurements of brevetoxins and their metabolites in certain substrates to nanogram levels (Poli et al. 2000), making it possible to isolate pure brevetoxins (including the different subtypes and metabolites) for use in the laboratory (Bourdelais et al. 2004; Pierce and Kirkpatrick 2001).

Because of beachgoer complaints about symptoms during red tides, researchers investigated the relationships between the presence of *K. brevis* and brevetoxins in marine ecosystems and the generation of toxin-containing aerosols at the seawater–air interface (Pierce et al. 1989, 1990). Newly developed analytical capabilities were applied to studies of exposure and subsequent body burdens in both laboratory animals and marine mammal, fish, and bird die-offs in the wild that were associated with Florida red tides and their aerosols (Bossart et al. 1998; Naar et al. 2002).

Recent laboratory investigations revealed the possibility of systemic chronic health effects (e.g., immunologic and neurologic) from inhaled, not just oral, brevetoxins (Benson et al. 1999). The researchers exposed 12-week-old male F344/Crl BR rats to a single exposure of 6.6 μg/kg PbTx-3 (brevetoxin 3) through intratracheal instillation. The study showed that more than 80% of the PbTx-3 was rapidly cleared from the lung and distributed by the blood throughout the body, with deposition in the brain. Although most of the PbTx-3 was excreted within 48 hr after exposure, the authors concluded that potential health effects associated with inhaled brevetoxins could extend beyond the reportedly transient respiratory irritation reported by humans exposed to Florida red tide brevetoxin aerosol to possible systemic chronic health effects.

Bossart et al. (1998) and Trainer and Baden (1999) extended their research beyond laboratory animal models to study the effect of *K. brevis* and brevetoxins on Florida wildlife. In 1996 a prolonged Florida red tide resulted in the deaths of 149 endangered Florida manatees. The manatees’ exposure to brevetoxins included chronic inhalation of the red tide toxin aerosol and ingestion of contaminated seawater over several weeks. Using a newly developed immunohistochemical stain, the researchers found that brevetoxins accumulated in the respiratory tract, liver, kidneys, and brains of the manatees and concluded that brevetoxin exposure contributed to the manatee deaths.

## Research Challenges

The iterative, synergistic research efforts to discover the underlying biology and biochemistry of *K. brevis* and aerosolized Florida red tide have provided a rich scientific foundation for new research in the fields of biology, chemistry, neurology, biochemistry, toxicology, oceanography, environmental sampling, aerosols, pathology, and public health. For example, the patterns of respiratory irritation seen in people who have inhaled aerosolized red tide toxins are consistent with the dynamics of ocean aerosol generation and the biologic activities of brevetoxins (Pierce et al. 1989, 1990). However, more data are needed about environmental exposures and the biologic activities of the red tide aerosols in living organisms.

One major challenge facing the interdisciplinary aerosolized Florida red tide research group is the characterization of the red tide aerosols in terms of biologically active constituents. This characterization must include the *K. brevis* cellular debris, the specific relative concentrations of brevetoxins, the newly identified brevetoxin antagonists (brevenal and its derivatives) (Bourdelais et al. 2004), and associated microorganisms. We also need to better understand the role of environmental conditions in producing red tide aerosols, including shoreline wind speed and direction, the possible effects of ultraviolet light, and the impact of wave activity on cellular integrity.

Our current analytical limits of detection present another challenge from the public health perspective. Extensive human populations are exposed to a potent neurotoxin aerosol that affects human health in doses that are at or near our analytic limits of detection (picogram to nanogram range). We suspect that people who have underlying illnesses and/or previous exposures that make them sensitive to respiratory irritants are particularly at risk from these exposures.

## This Mini-Monograph

The articles included in this mini-monograph describe the most recent activities of our ongoing interdisciplinary research program to characterize aerosolized brevetoxin exposures and to evaluate their effects in animal models and humans. The Florida red tide aerosol research program represents an interdisciplinary and interagency collaboration that has built upon, and expanded, existing knowledge. Within the last 5 years, these collaborative efforts have resulted in *a*) identification of brevenal, a new brevetoxin antagonist elaborated by *K. brevis* itself; *b*) development of a highly sensitive brevetoxin ELISA, as well as refinement of existing analytical techniques, applicable to multiple substrates; *c*) characterization of the particle size distribution of the Florida red tide aerosol; *d*) measurement of complex mixtures of brevetoxin levels in ambient and personal breathing zone air; *e*) exploration of acute and chronic effects of the Florida red tide aerosols in rodent models and in an asthmatic sheep model; *f* ) expansion of the understanding of the toxicology and physiology of the brevetoxins; *g*) initial evaluation of self-reported symptoms and objective measurements of physiologic respiratory changes in humans with recreational and occupational exposures; and *h*) initial identification of readily available medications to prevent and/or treat the effects of exposure to the Florida red tide aerosols (Abraham et al. 2005; Backer et al. 2005; Baden et al. 2005; Benson et al. 2005; Bourdelais et al. 2004; Cheng et al. 2005; Fleming et al. 2005; Naar et al. 2002; Pierce et al. in press)

The interdisciplinary synergy within the red tide research group has generated considerable progress in understanding the exposures and potential impacts of Florida red tide aerosols. We are advancing toward our goal: understanding whether exposure to these aerosols represents a public health threat beyond the nuisance eye, nose, and throat irritation, and determining whether people who have underlying respiratory conditions such as asthma, should take special precautions when visiting the beach during a red tide. Integrating this research program with ongoing oceanographic monitoring and modeling will help to establish Florida red tide early warning systems to mitigate or even prevent exposure in susceptible human populations (both residents and tourists). Finally, even though we have specifically applied the expertise in this research program to investigate Florida red tide aerosols, our approach is a model for the study of other HAB-related toxins and for other aerosolized environmental contaminants.
